# Supplementary material for: Brain Segregation and Integration Relate to Word-Finding Abilities in Older and Younger Adults
Source: Neurobiol Lang (Camb). 2025 Jul 15;6:nol.a.7. doi: 10.1162/nol.a.7 (PMC12307025; doi:10.1162/nol.a.7)
Supplement: Supplementary file 1 [file nol-6-1-7-s001.pdf]

Supplementary Materials

S1. A-Priori Power Analysis for Sample Size

A-priori power analysis using data simulation was used to confirm power for the final sample size (Brysbaert & Stevens, 2018; DeBruine & Barr, 2019). Because this study uses secondary data analysis, the number of participants in this study was already known. Therefore, the sample size was held constant in the power analysis. For the analysis, we estimated means and standard deviations for the verbal fluency based on existing literature (Aita et al., 2019; Bäckman & Nilsson, 1996; Gibson et al., 2019; Gonzalez-Burgos et al., 2019; Sokołowski et al., 2020) and EEG parameters (Feklicheva et al., 2021; Gaál et al., 2010; Utianski et al., 2016) that closely resembled our study design (DeBruine & Barr, 2021). For the power analyses, we simulated the data set with verbal fluency and functional connectivity values, which were randomly generated with a normal distribution centred around the mean, for 1000 runs using a script in RStudio (DeBruine & Barr, 2021). Multiple linear regression models were defined within a function and performed for each simulation run. We used R (R Core Team, 2020) to simulate the data and to run the statistical models. Following the method outlined by DeBruine & Barr (2019), power was defined as mean (p value < alpha), where alpha was set at 0.05. We obtained average to high power (60-90%) for our models depending on the frequency band and the EEG measure, and hence, concluded that our sample size is sufficient for our analyses. For more information on the power analysis, please see the pre-registration.

S2. Functional connectivity measures

Debiased weighted Phase Lag Index

Oscillatory phase (which is provided by the EEG data) can be visualised with a circular axis, where the x-axis is called the real axis and the y-axis the imaginary axis. Imaginary numbers on the imaginary axis are necessary when we cannot use the real number system (i.e., the numbers we use in our day-to-day life) to solve a mathematical problem, for example, when taking the square root of a negative number. Complex numbers are the sum of a real and an imaginary number, and are necessary to obtain information, such as phase and power information from EEG data (Cohen, 2014). The dwPLI measures phase synchrony, whilst reducing the effect of volume conduction by ignoring 0 or π phase differences between electrodes, which reflect instantaneous synchronisation. These instantaneous synchronisations are physiologically implausible as markers of neural synchronisation through synaptic transmission (however, see Hajizadeh et al., 2022 for a biologically plausible mechanism of instantaneous synchronisation) and are more likely to reflect volume conduction of electrical potentials to both electrodes simultaneously (Vinck et al., 2011). As such, the dwPLI measures how much a signal of one channel leads or lags compared to the signal of another channel and is weighted by the magnitude of the imaginary component of the cross-spectrum (i.e., the cross-correlation between two timeseries). The formula to compute the dwPLI is as follows (Vinck et al., 2011):

$$\hat{\Omega}\text{w}\ (f) = \frac{\sum_{j=1}^{N}\sum_{k\neq j} Im\{X_j\}Im\{X_k\}}{\sum_{j=1}^{N}\sum_{k\neq j} |Im\{X_j\}Im\{X_k\}|},$$

where $\hat{\Omega}\text{w}\ (f)$ is the dwPLI at frequency *f* and *N* is the total number of epochs. The numerator is the sum of the imaginary component of all pairwise products of the cross-spectra X between epoch *j* and epoch *k*, which is the computation of between-electrode connectivity, reflect the same time epoch, but epochs extracted from two different electrodes. The denominator is the sum of all pairwise products of the magnitude of the imaginary component between epoch *j* and epoch *k*. The computation of the dwPLI preserves the sign (i.e., whether the denominator is positive or negative).

Orthogonal Minimum Spanning Trees

We applied a data-driven method, namely the Orthogonalized Minimum Spanning Tree (OMST) algorithm to threshold the connectivity matrices (Dimitriadis et al., 2017). A minimum spanning tree is a graph with a minimum number of total edges, without cycles (i.e., the graph does not contain any loops), and where all nodes are connected (see Figure 1C). The OMST algorithm computes the minimum spanning tree (MST) over multiple iterations. These are necessary because it is possible that by using only a single MST, one ends up with a graph that is too sparse for computing robust connectivity measures. After each iteration, the edges (N-1) of the minimum spanning tree in that iteration are set to infinity so the algorithm ignores those connections in the subsequent iteration. Because the OMST algorithm computes multiple spanning trees to threshold the connectivity matrices for further analyses, it preserves as many connections as possible (i.e., reducing wiring cost, where adding connections come at the expense of the networks performance or efficiency), whilst maintaining an optimal global efficiency of the network. The OMSTs are calculated from the inverse weighted graph, which can be calculated from the connectivity matrix. In an inverse weighted graph, the strongest connections represent the brain areas that are functionally closer or more strongly connected.

Weighted Clustering Coefficient

The weighted clustering coefficient (C^W), expresses how frequently, on average, a node is part of a local triangle of connections. That is, how often when a node is connected to one of its neighbours, these neighbours are also connected to each other. The formula of the weighted clustering coefficient is as follows (Onnela et al., 2005; Rubinov & Sporns, 2010):

$$C^W = \frac{1}{n}\sum_{i \in N} \frac{2t_i^w}{k_i(k_i - 1)},$$

where i is the node index, n is the total number of nodes in graph N, and k_i^w represents the summed weight of all edges that are connected to that node (i.e., weighted degree of node i). t^w is the geometric mean of the triangles' weight at node i. Finally, the clustering coefficient is calculated by averaging the clustering coefficient at all nodes.

Modularity

Modularity measures the prevalence of non-overlapping modules or sub-networks in a graph according to (Newman, 2006; Rubinov & Sporns, 2010):

$$Q^W = \frac{1}{l^W}\sum_{i,j\in N}\left[w_{ij} - \frac{k_i^w k_j^w}{l^w}\right]\delta_{m_i m_j},$$

where Q^W is the weighted modularity. L^W is the sum of all weights in graph N, and w_{ij} is the undirected weight of the edge between node i and j. k_i^w is the weighted degree of node i. m_i is the module containing node i and where the delta function $\delta_{m_i m_j}$ is 1 if $i = j$ and 0 otherwise. The maximum value of Q^W is 1, which means that the network is strongly divided into communities (i.e., significantly more intra-community connections compared to inter-community connections).

Characteristic Path Length

The formula of the characteristic path length is as follows (Rubinov & Sporns, 2010; Watts & Strogatz, 1998):

$$L^W = \frac{1}{n}\sum_{i\in N}\frac{\sum_{j\in N, j\neq i} d_{ij}^W}{n-1}$$

where L^W is the weighted characteristic path length. To calculate d_{ij}^W, the weights of the edges are converted to length by calculating the reciprocal of the weights (1/weight) in the matrix (i.e., taking the inverse of the weighted matrix) to reflect the distance between node i and j

when there is a connection between i and j. Stronger connections represent stronger associations between node i and j and a shorter distance.

Small-World Index

To examine the balance between brain segregation and integration, we computed the small-world index of each graph. First, we created random networks by randomly swapping (100 times) the edge weights of the original networks, whilst preserving the weight, degree, and strength distribution (Rubinov & Sporns, 2010). Next, we calculated the clustering coefficient (C^W_{rand}) and characteristic path length (L^W_{rand}) of the random networks. Small-worldness was obtained through the following formula (Humphries & Gurney, 2008):

$$S^W = \frac{C^W / C^W_{rand}}{L^W \,/ L^W_{rand}}$$

where a value higher than 1 indicates that the network is a small world.

S3. The Relationship between Characteristic Path Length and Modularity

Our study showed an inverse between alpha band modularity and semantic fluency scores, irrespective of age. In our discussion, we proposed that brain integration might be more important for semantic fluency performance than brain segregation and it is possible that greater brain segregation could be related to lower integration. Hence, we hypothesised an inverse relationship between brain integration (characteristic path length) and brain segregation (modularity) in the alpha band. That is, if brain integration is more important than segregation for semantic fluency, the relationship between greater modularity and lower semantic fluency scores could potentially be explained by a lower characteristic path length.

To investigate this idea, we conducted a multiple linear regression analysis with the OMST-thresholded characteristic path length as the outcome variable and the OMST-thresholded modularity as the predictor. To keep in line with our previous models, we added

Sex (2 levels) as covariate. Leverage points were removed accordingly. We did not include Age as a predictor or covariate as the relationship between modularity and semantic fluency did not interact with age. After the initial model fit, leverage points were identified as 2(number of predictors + 1)/number of observations, and subsequently removed to obtain the model's best fit. The predictors explained 13.5% of the variance in semantic fluency scores ($R^2 = .12$, $F(2,96) = 7.50$, $p = .001$). Modularity was a significant predictor of the characteristic path length ($\beta = 0.60$, $p < .001$). Hence, in the alpha band, when brain segregation is higher, brain integration is also higher (see Figure S3.1).

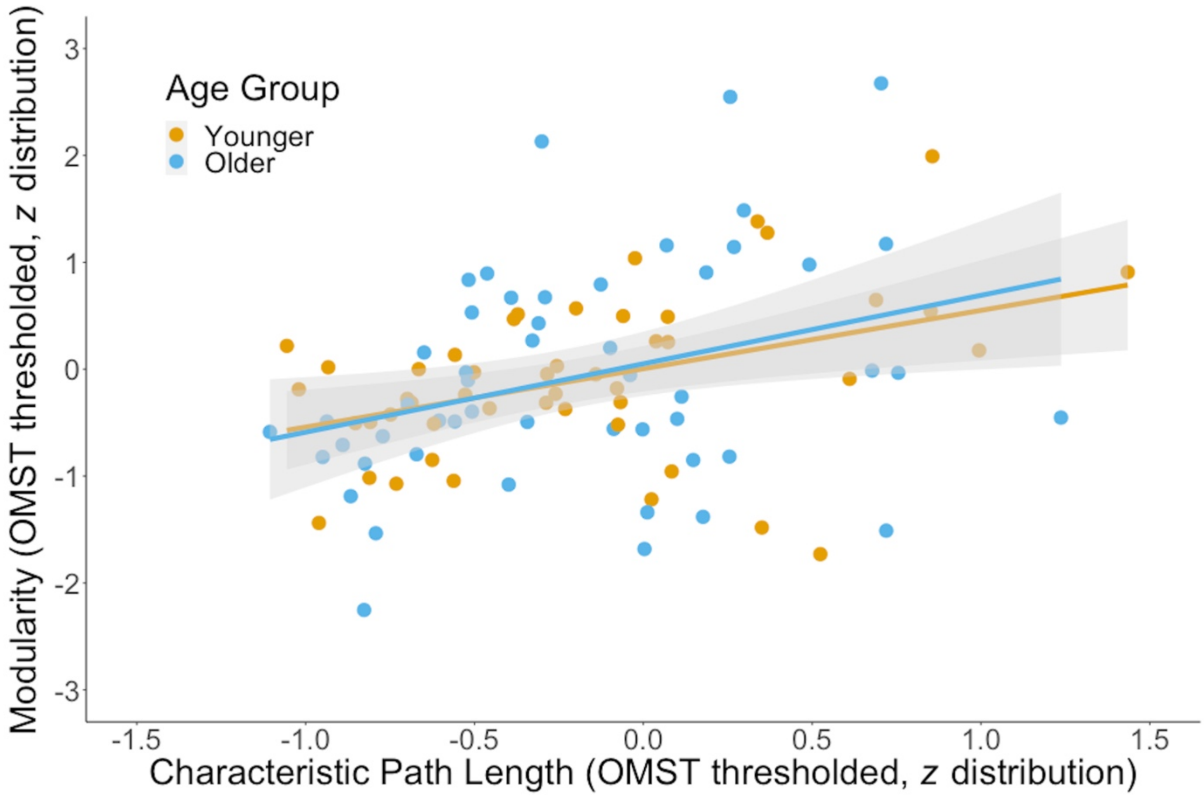

Figure S3.1 The relationship between modularity (brain segregation) and characteristic path length (brain integration)

References

Aita, S. L., Beach, J. D., Taylor, S. E., Borgogna, N. C., Harrell, M. N., & Hill, B. D. (2019). Executive, language, or both? An examination of the construct validity of verbal fluency measures. *Applied Neuropsychology: Adult*, *26*(5), 441-451. https://doi.org/10.1080/23279095.2018.1439830

Bäckman, L., & Nilsson, L.-G. (1996). Semantic memory functioning across the adult life span. *European Psychologist*, *1*(1), 27-33. https://doi.org/10.1027/1016-9040.1.1.27

Brysbaert, M., & Stevens, M. (2018). Power Analysis and Effect Size in Mixed Effects Models: A Tutorial. *Journal of Cognition*, *1*(1), Article 1. https://doi.org/10.5334/joc.10

Cohen, M. X. (2014). *Analyzing neural time series data: Theory and practice*. The MIT Press.

DeBruine, L. M., & Barr, D. J. (2019). *Understanding mixed effects models through data simulation* [Preprint]. PsyArXiv. https://doi.org/10.31234/osf.io/xp5cy

DeBruine, L. M., & Barr, D. J. (2021). Understanding Mixed-Effects Models Through Data Simulation. *Advances in Methods and Practices in Psychological Science*, *4*(1), 2515245920965119. https://doi.org/10.1177/2515245920965119

Dimitriadis, S. I., Salis, C., Tarnanas, I., & Linden, D. E. (2017). Topological Filtering of Dynamic Functional Brain Networks Unfolds Informative Chronnectomics: A Novel Data-Driven Thresholding Scheme Based on Orthogonal Minimal Spanning Trees (OMSTs). *Frontiers in Neuroinformatics*, *11*, 28. https://doi.org/10.3389/fninf.2017.00028

Feklicheva, I., Zakharov, I., Chipeeva, N., Maslennikova, E., Korobova, S., Adamovich, T., Ismatullina, V., & Malykh, S. (2021). Assessing the Relationship between Verbal and

Nonverbal Cognitive Abilities Using Resting-State EEG Functional Connectivity. *Brain Sciences*, *11*(1), 94. https://doi.org/10.3390/brainsci11010094

Gaál, Z. A., Boha, R., Stam, C. J., & Molnár, M. (2010). Age-dependent features of EEG-reactivity—Spectral, complexity, and network characteristics. *Neuroscience Letters*, *479*(1), 79-84. https://doi.org/10.1016/j.neulet.2010.05.037

Gibson, E. C., Barker, M. S., Martin, A. K., & Robinson, G. A. (2019). Initiation, Inhibition and Strategy Generation Across the Healthy Adult Lifespan. *Archives of Clinical Neuropsychology: The Official Journal of the National Academy of Neuropsychologists*, *34*(4), 511-523. https://doi.org/10.1093/arclin/acy057

Gonzalez-Burgos, L., Hernández-Cabrera, J. A., Westman, E., Barroso, J., & Ferreira, D. (2019). Cognitive compensatory mechanisms in normal aging: A study on verbal fluency and the contribution of other cognitive functions. *Aging*, *11*(12), 4090-4106. https://doi.org/10.18632/aging.102040

Humphries, M. D., & Gurney, K. (2008). Network 'Small-World-Ness': A Quantitative Method for Determining Canonical Network Equivalence. *PLOS ONE*, *3*(4), e0002051. https://doi.org/10.1371/journal.pone.0002051

Newman, M. E. J. (2006). Modularity and community structure in networks. *Proceedings of the National Academy of Sciences*, *103*(23), 8577-8582. https://doi.org/10.1073/pnas.0601602103

Onnela, J.-P., Saramäki, J., Kertész, J., & Kaski, K. (2005). Intensity and coherence of motifs in weighted complex networks. *Physical Review E*, *71*(6), 065103. https://doi.org/10.1103/PhysRevE.71.065103

R Core Team. (2020). *R: A Language and Environment for Statistical Computing* [Software]. R Foundation for Statistical Computing. https://www.R-project.org/

Rubinov, M., & Sporns, O. (2010). Complex network measures of brain connectivity: Uses and interpretations. *NeuroImage*, *52*(3), 1059-1069. https://doi.org/10.1016/j.neuroimage.2009.10.003

Sokołowski, A., Tyburski, E., Sołtys, A., & Karabanowicz, E. (2020). Sex Differences in Verbal Fluency Among Young Adults. *Advances in Cognitive Psychology*, *16*(2), 92-102. https://doi.org/10.5709/acp-0288-1

Utianski, R. L., Caviness, J. N., van Straaten, E. C. W., Beach, T. G., Dugger, B. N., Shill, H. A., Driver-Dunckley, E. D., Sabbagh, M. N., Mehta, S., Adler, C. H., & Hentz, J. G. (2016). Graph theory network function in Parkinson's disease assessed with electroencephalography. *Clinical Neurophysiology*, *127*(5), 2228-2236. https://doi.org/10.1016/j.clinph.2016.02.017

Vinck, M., Oostenveld, R., van Wingerden, M., Battaglia, F., & Pennartz, C. M. A. (2011). An improved index of phase-synchronization for electrophysiological data in the presence of volume-conduction, noise and sample-size bias. *NeuroImage*, *55*(4), 1548-1565. https://doi.org/10.1016/j.neuroimage.2011.01.055

Watts, D. J., & Strogatz, S. H. (1998). Collective dynamics of 'small-world' networks. *Nature*, *393*(6684), Article 6684. https://doi.org/10.1038/30918
